# Influence of Temperature on the Mechanical Behavior of Lead/Rubber Bearings

**DOI:** 10.3390/polym18111306

**Published:** 2026-05-26

**Authors:** Fan Yang, Lixiu Zhang, Hui Pang, Tao Jiang

**Affiliations:** 1 School of Civil Engineering & Transportation, Bei Hua University, Jilin 132013, China; yangfan19821121@163.com; 2 CCCC Second Harbour Engineering Jilin Construction Co., Ltd., Changchun 130000, China; 13634414972@163.com; 3 Key Laboratory of Earthquake Engineering and Engineering Vibration, Institute of Engineering Mechanics, China Earthquake Administration, Harbin 150080, China; m18686805613@163.com; 4 Key Laboratory of Earthquake Disaster Mitigation, Ministry of Emergency Management, Harbin 150080, China

**Keywords:** lead/rubber bearing, ambient temperature, hysteretic heating, constitutive model, seismic response

## Abstract

The mechanical behavior of lead/rubber bearings (LRBs) is strongly influenced by both ambient temperature and hysteretic heating under seismic loading; however, their coupled effects and underlying mechanisms remain insufficiently understood. This study presents a systematic investigation of the thermo-mechanical response of LRBs through combined experimental and numerical approaches. Dynamic cyclic tests were conducted on full-scale LRBs (700 mm in diameter) over a wide range of ambient temperatures, revealing that ambient temperature and hysteretic heating jointly govern the evolution of key mechanical properties, including stiffness, characteristic strength, and energy dissipation capacity. Specifically, decreasing temperature leads to stiffness and strength enhancement, whereas hysteretic heating induced by cyclic plastic deformation of the lead core results in progressive softening and degradation of restoring force. Based on the experimental observations, a modified uniaxial Bouc–Wen constitutive model is developed, incorporating the coupled effects of ambient temperature, hysteretic heating, and large-strain hardening. The proposed model is implemented in a single-degree-of-freedom (SDOF) base-isolated system to evaluate the seismic response under different temperature conditions. The results reveal a competing mechanism between ambient temperature and hysteretic heating: low temperatures tend to increase base shear and reduce displacement, while hysteretic heating produces the opposite effect, with their relative dominance depending on temperature level and ground motion intensity. Neglecting such thermo-mechanical coupling may lead to significant misestimation of structural response, particularly under long-duration strong ground motions. This study provides new insights into the coupled temperature-dependent behavior of LRBs and establishes a robust modeling framework for the seismic analysis and design of isolation systems under complex service conditions.

## 1. Introduction

Seismic isolation technology reduces earthquake-induced structural responses by introducing a flexible isolation layer between the superstructure and the foundation. This effectively lengthens the fundamental period of the structure, avoids resonance with dominant ground motion periods, and significantly reduces seismic energy transfer, thereby mitigating structural damage [1,2,3]. Over the past decades, various isolation devices have been developed, including laminated rubber bearings, sliding bearings, and friction pendulum systems. Among these, laminated rubber bearings are the most widely used due to their simple configuration, stable performance, and reliability. Depending on material composition and energy dissipation mechanisms, they are classified into natural rubber bearings, lead/rubber bearings (LRBs), and high-damping rubber bearings. These bearings consist of alternating layers of rubber and thin steel plates, providing high vertical load capacity while allowing large horizontal deformations.

In seismic isolation systems, the isolation layer serves as a controllable flexible component that governs the structural seismic response. As its core element, the performance of rubber bearings and their accurate representation in design are critical to structural safety and reliability. Although various models, such as bilinear and Bouc–Wen formulations, have been developed and widely applied [4,5,6], existing studies have extensively conducted parameter sensitivity analysis of hysteretic models represented by the Bouc–Wen model to quantify parameter uncertainty and its influence on structural response. Worden and Beck [7] established a Bayesian framework for the sensitivity analysis and parameter confidence assessment of hysteretic systems, which provides a solid probabilistic foundation for evaluating the parameter uncertainty of the Bouc–Wen model. Complementarily, Haukaas and Jeremic [8] proposed an exact sensitivity analysis approach based on tangent stiffness formulation for nonlinear dynamic responses of hysteretic systems including the Bouc–Wen model, which can accurately quantify the influence of parameter variation on the seismic performance of base-isolated structures. Drawing on the above classical theoretical methods, this paper carries out systematic parameter sensitivity analysis for the Bouc–Wen model, so as to improve the rationality and reliability of numerical modeling of lead/rubber bearings. Key factors affecting bearing behavior remain insufficiently understood. Among these, temperature effects are particularly important. Ambient temperature significantly influences both rubber and lead properties: at low temperatures, rubber stiffens and becomes more brittle, while the yield strength of the lead core increases, resulting in higher overall stiffness and altered energy dissipation, which may degrade the actual mechanical performance. In addition, under cyclic loading, plastic deformation of the lead core generates heat (hysteretic heating), leading to a temperature rise that reduces characteristic strength and stiffness. This, in turn, weakens the restoring force, modifies hysteretic behavior, and may adversely affect the performance of isolation devices and the safety of structures under strong seismic events.

Experimental studies consistently show that ambient temperature significantly affects key mechanical parameters of rubber bearings, including vertical and horizontal stiffness and characteristic strength. Tests conducted over a wide temperature range (approximately −40 °C to 50 °C) indicate that decreasing temperature leads to increased rubber stiffness, fuller hysteresis loops, and enhanced energy dissipation [9,10,11]. However, the resulting stiffness hardening may amplify seismic responses of isolated structures, as confirmed by observed damage in cold regions [12,13,14]. To address temperature effects, several studies have proposed temperature-dependent correction functions and models based on experimental data [15,16,17,18,19]. These findings are further supported by Constantinou et al. [20], which have been incorporated into design provisions such as the AASHTO [21]. Overall, ambient temperature is a critical factor influencing both the mechanical behavior of rubber bearings and the seismic response of isolated structures, with low-temperature effects posing notable risks to structural safety.

In addition to ambient temperature effects, lead/rubber bearings (LRBs) exhibit a pronounced internal temperature rise under cyclic loading. During repeated shear deformation, plastic work in the lead core is converted into heat, leading to rapid temperature increase, commonly referred to as the hysteretic heating effect [22,23,24]. This thermo-mechanical coupling causes time-dependent degradation of material properties, as increasing temperature reduces the yield strength and stiffness of the lead core, resulting in softened hysteretic behavior, weakened restoring force, and diminished energy dissipation capacity. Compared with analyses considering only ambient temperature, accounting for hysteretic heating provides a more realistic representation of the performance evolution of LRBs under strong seismic loading.

Extensive studies on hysteretic heating have been conducted using theoretical, experimental, and numerical approaches. Early work based on energy conservation showed that lead/rubber bearings (LRBs) dissipate seismic energy primarily through heat generated by plastic deformation of the lead core [25,26,27]. Experiments by Xue et al. [28] demonstrated that cyclic shear can raise the lead core temperature to 150–200 °C, significantly reducing characteristic strength and stiffness, and decreasing hysteresis loop area. Shiraz [29] further clarified that the generated heat dissipates through end plates and steel layers, forming a complex temperature field. Subsequent research has confirmed the substantial impact of hysteretic heating on seismic response [30]. Temperature rise weakens restoring force and modifies hysteretic behavior, affecting displacement demand and energy dissipation [31,32]. Under near-fault motions or large cyclic deformations, this effect becomes more pronounced and may amplify structural response or increase collapse risk [33,34]. To address this, temperature-dependent bilinear models and equivalent linearization methods have been proposed, improving simulation accuracy to some extent [35,36,37,38,39].

Despite extensive studies on ambient temperature and hysteretic heating effects, several limitations remain. First, most experiments are conducted on scaled or simplified specimens, whose geometry and heat dissipation differ from full-scale bearings, limiting the accurate representation of temperature evolution; notably, full-scale test data are insufficient, which further hinders the reliability and applicability of the research findings, as scaled specimens cannot fully replicate the actual service conditions and mechanical responses of full-scale lead/rubber bearings. Second, relatively low loading frequencies are often adopted, which cannot fully reproduce the high-frequency, large-amplitude cyclic loading during earthquakes, potentially underestimating hysteretic heating in lead cores; additionally, research on the behavior under long-duration earthquakes is limited, and the thermo-mechanical response of bearings under prolonged cyclic loading—one of the key scenarios in actual seismic events—has not been adequately explored. Third, although ambient temperature and hysteretic heating jointly influence bearing behavior, they are typically studied separately, and a unified framework capturing their coupled effects—from initial temperature to cyclic heating and subsequent degradation—is still lacking; more importantly, research on coupled temperature effects is inadequate, as the mutual interaction and synergistic influence mechanism between ambient temperature and hysteretic heating have not been thoroughly clarified. Finally, existing constitutive models generally treat temperature effects in a simplified manner and fail to simultaneously account for the coupling between temperature dependence and cyclic degradation; in other words, the existing constitutive models are overly simplified, as they cannot accurately characterize the dynamic evolution of bearing mechanical properties under the combined action of temperature variation and cyclic loading, nor can they effectively reflect the cumulative degradation effect caused by long-term thermo-mechanical coupling.

In light of the above, this study investigates the variation in mechanical behavior of lead/rubber bearings (LRBs) induced by ambient temperature and hysteretic heating under long-duration strong ground motions. Dynamic cyclic tests are performed on full-scale LRBs (700 mm diameter) under different ambient temperatures, with temperature field monitoring used to quantify the coupled effects of external temperature and internal heating. Based on the results, a uniaxial constitutive model is developed, incorporating ambient temperature, hysteretic heating, and large-strain hardening. Seismic response analyses of a single-degree-of-freedom (SDOF) base-isolated system are then conducted to evaluate the influence of coupled temperature effects. This study provides a reliable basis for the performance assessment and engineering application of rubber bearings under complex service conditions.

## 2. Temperature-Dependent Dynamic Tests of the LRB700

### 2.1. Lead/Rubber Bearing Specimens

**Figure 1:**
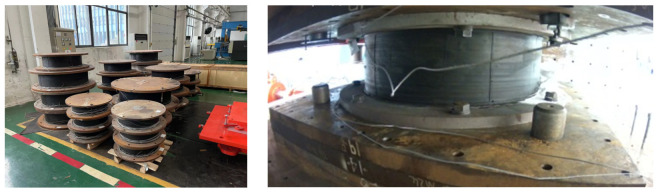
Photos of the LRB700 specimen.

Three typical lead/rubber bearings (LRBs) with a diameter of 700 mm were selected in this study (see Figure 1), denoted as LRB700-1, LRB700-2, and LRB700-3. The detailed geometric dimensions of the bearings are summarized in Table 1. The lead/rubber bearings adopted in this study are all provided by Feng ze Intelligent Equipment Co., Ltd. (No. 15 Xiangsu Road, Northern Industrial Base, Heng shui Economic Development Zone, Hebei Province, China). The relevant performance parameters are offered by the manufacturer, and these parameters can be further referenced in Pang [40].

### 2.2. Dynamic Loading Tests

**Figure 2:**
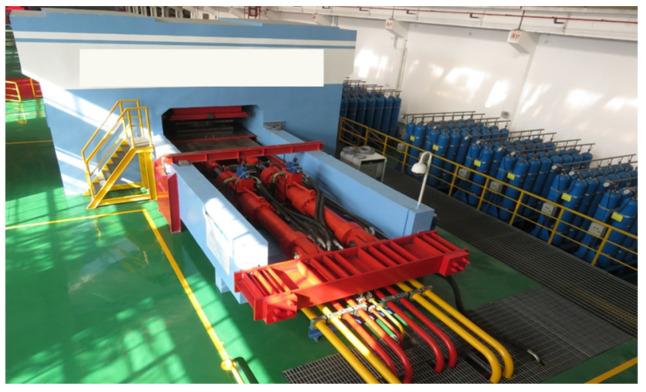
Setup of the loading system.

**Figure 3:**
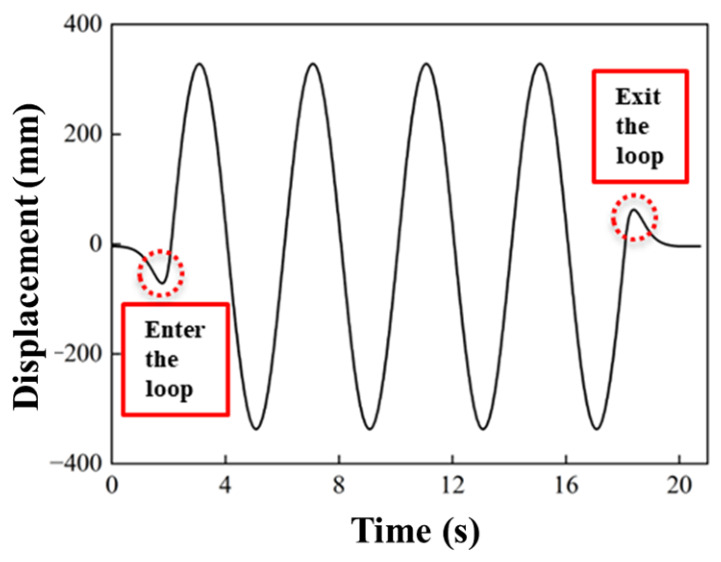
Loading regime.

The temperature-dependent tests on lead/rubber bearings were conducted using a multifunctional loading system, as shown in Figure 2, with the loading protocol illustrated in Figure 3. The detailed procedure can be found in Pang [40]. A total of 27 test conditions were designed, which are summarized in Table 2. According to GB/T 51408-2021 [41], the vertical load was applied as 15 MPa, corresponding to the standard design limit for the bearings, and the loading frequency was set to 0.20 Hz. According to GB 50011–2010 [42], shear strain amplitudes of 50%, 100%, and 250% were adopted, representing service-level, design-level, and maximum considered earthquake conditions, respectively. In addition, based on relevant bearing specifications, the temperature conditions were set at −20 °C, 0 °C, and 23 °C.

### 2.3. Cooling Process of the LRB700 Specimen

**Figure 4:**
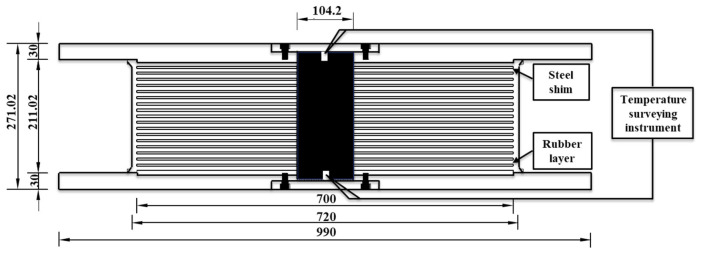
Configurations of the LRB700 specimen and the location of the thermocouple (unit: mm).

**Figure 5:**
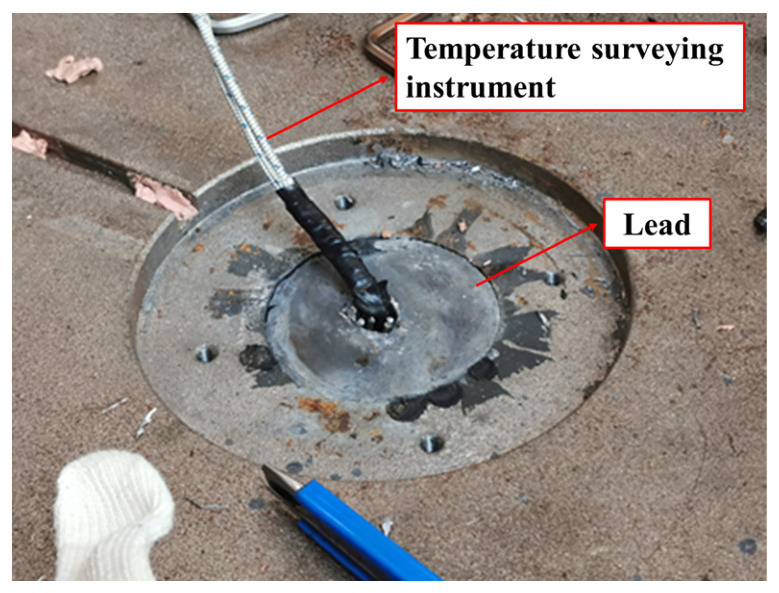
Arrangement drawing of thermocouples for the LRB700 specimen.

Prior to conducting the high-speed compression–shear tests at −20 °C and 0 °C, the specimens were preconditioned to the target temperatures. Before cooling, four groups of thermocouples were installed within the lead core to monitor internal temperature variations. All thermocouples were embedded to a depth of 5 cm from the inner surface of the end plate. The configuration of the bearing and the arrangement of measurement points are shown in Figure 4, while a detailed layout of thermocouples within the lead/rubber bearing is illustrated in Figure 5.

**Figure 6:**
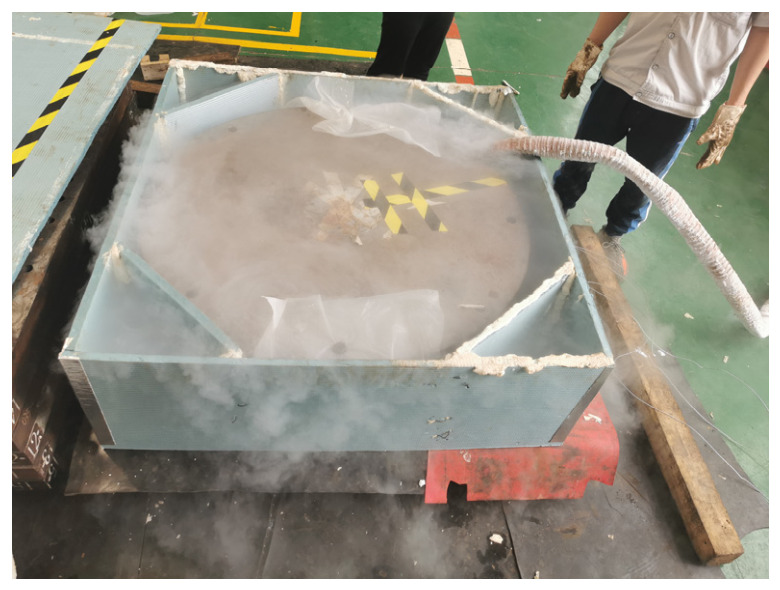
Cooling process of specimen.

The procedure for the low-temperature dynamic tests is as follows:

1. Cooling was achieved by continuously supplying dry ice, allowing the low temperature to be transferred into the bearing through the top and bottom end plates, as shown in Figure 6.
2. The bearing was then placed in a custom-made insulated chamber (see Figure 6). Once the thermocouple readings dropped slightly below the target test temperature (to account for temperature rise during handling and installation), the specimen was kept under insulation for an additional 1–2 h to reach thermal equilibrium. The specimen was subsequently installed rapidly into the multifunctional testing system, and the compression–shear test was initiated immediately.
3. During testing, a constant vertical compressive stress of 15 MPa was applied. Cyclic horizontal loading was imposed under sinusoidal displacement control, with shear strain amplitudes of 50%, 100%, and 250%. Hysteretic behavior was evaluated over 4–30 loading cycles.
4. After each test condition, the bearing was left at rest for 24 h, then reconditioned in the insulated chamber to the next target temperature, and Steps 2–4 were repeated.

### 2.4. Hysteresis Loops of the Specimens

**Figure 7:**
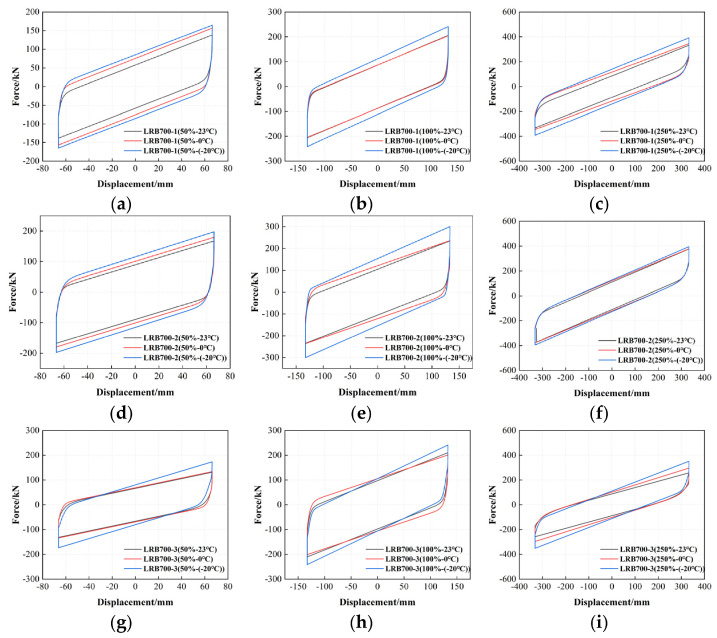
Hysteresis loops of LRB700 at different ambient temperatures and shear strain amplitudes (**a**) LRB700-1-50%; (**b**) LRB700-1-100%; (**c**) LRB700-1-250%; (**d**) LRB700-2-50%; (**e**) LRB700-2-100%; (**f**) LRB700-2-250%; (**g**) LRB700-3-50%; (**h**) LRB700-3-100%; (**i**) LRB700-3-250%.

By adopting the correction method proposed by Pang [43] for inertial and frictional effects of the loading system, the hysteretic behavior of rubber bearings under various loading conditions can be accurately obtained. According to GB/T 20688.1-2007 [41], the mechanical properties of rubber bearings are determined based on either the third loading cycle or the average response from the second to the eleventh cycles in repeated cyclic tests. To examine the variation of mechanical properties with ambient temperature, the hysteresis loop from the third cycle is adopted as the reference in this study. Figure 7 presents the hysteretic curves of the rubber bearings under a vertical compressive stress of 15 MPa at different ambient temperatures where blue, red, and black lines correspond to −20 °C, 0 °C, and 23 °C, respectively.

As shown in Figure 7, the hysteresis loops of LRB700-1, LRB700-2, and LRB700-3 exhibit significant differences under varying ambient temperatures, indicating that the mechanical behavior of rubber bearings is strongly temperature-dependent. It is obvious that the hysteresis loop area of the LRB700 bearings increases with decreasing temperature.

## 3. Temperature-Dependent Mechanical Behavior of the LRB700 Specimen

**Figure 8:**
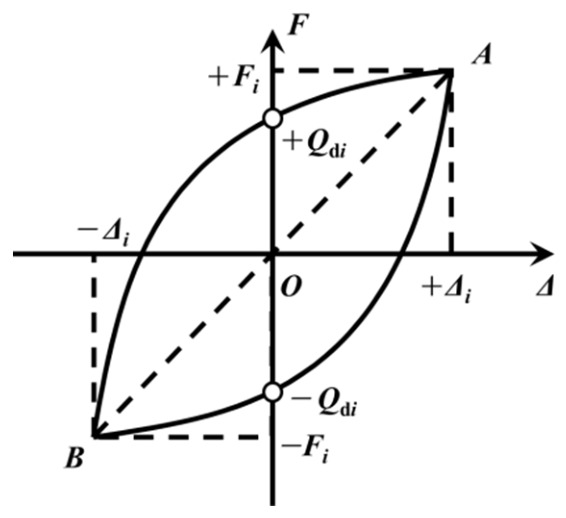
Schematic of the characteristic strength and equivalent stiffness calculation.

As per the specifications outlined in GB/T 20688.1-2007 [41], the characteristic strength of a lead/rubber bearing is determined by calculating the average absolute value of the *y*-axis intercept, as depicted in Figure 8.

(1) $$Q_{d,i}=\frac{1}{2}(\left|+Q_{di}\right|+\left|-Q_{di}\right|)$$

where +*Q*_d*i*_ is the positive intercept of the *i*-th hysteresis curve and the *y*-axis, and −*Q*_d*i*_ is the negative intercept of the *i*-th hysteresis curve and *y*-axis.

The calculation for the horizontal equivalent stiffness of the rubber bearing is as follows:

(2) $$K_{h,i}=\frac{F_{i}-(-F_{i})}{\Delta_{i}-(-\Delta_{i})}$$

where *F_i_* and −*F_i_* are the restoring forces corresponding to the maximum and minimum displacements, respectively, in the *i*-th hysteresis loop of Figure 8.

**Figure 9:**
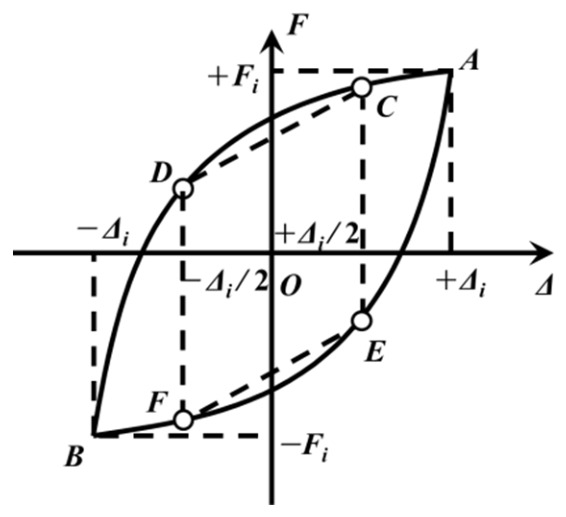
Schematic of the horizontal post-yield stiffness calculation.

The post-yield stiffness of a lead/rubber bearing can be ascertained using the following equation:

(3) $$K_{post,i}=\frac{1}{2}(\frac{F_{C}-F_{D}}{\Delta_{c}-\Delta_{D}}+\frac{F_{E}-F_{F}}{\Delta_{E}-\Delta_{F}})$$

where C, D, E, and F are the four points at the displacement of Δ*i*/2 and −Δ*i*/2 on the hysteresis curve in Figure 9, and Δ*i* and −Δ*i* represent the maximum and minimum shear displacements in the i-th hysteresis loop, respectively. Consequently, the post-yield stiffness is therefore defined as the mean gradient of the CD and EF lines.

Using Equations (1)–(3), the characteristic strength, post-yield stiffness, and horizontal equivalent stiffness of the LRB700 bearings can be determined from the third cycle at a shear strain amplitude of 100%. These mechanical properties are summarized in Table 3, Table 4 and Table 5.

Table 3 presents the variation of characteristic strength with temperature. It can be observed that the characteristic strength of the LRB700 bearings at −20 °C is significantly higher than that at 0 °C and 23 °C, indicating a pronounced temperature dependence. This behavior is primarily attributed to the crystallization hardening of the lead core material. As the temperature decreases from 23 °C to −20 °C, the yield strength of the lead core increases markedly, resulting in a 32% increase in the characteristic strength of the LRB700 bearings.

As shown in Table 4, the post-yield stiffness of the LRB700 bearings remains nearly unchanged with decreasing temperature. This phenomenon can be attributed to the fact that the post-yield stiffness of lead/rubber bearings is primarily governed by the rubber layers. Within the considered temperature range, the mechanical properties of rubber remain relatively stable, and its shear stiffness exhibits only minor variation, resulting in an almost temperature-independent post-yield stiffness.

Table 5 illustrates the variation of horizontal equivalent stiffness with temperature. It is observed that the equivalent horizontal stiffness of the LRB700 bearings at −20 °C is significantly higher than that at 0 °C and 23 °C. When the temperature decreases from 23 °C to −20 °C, the yield strength of the lead core increases significantly, leading to a 24% increase in the horizontal equivalent stiffness of the LRB700 bearing. This variation is mainly caused by the enhancement of the lead core yield strength at low temperature, which improves the restoring force of the bearing. Accordingly, under a constant displacement amplitude, the horizontal equivalent stiffness increases as the temperature decreases.

To account for the influence of ambient temperature on the mechanical performance of rubber bearings, Appendix D of GB/T 51408-2021 [41] introduces three modification factors, namely, the adjustment coefficient of characteristic strength, the adjustment coefficient of post-yield stiffness, and the adjustment coefficient of horizontal equivalent stiffness. These three factors are defined in Equations (4)–(6), to adjust the mechanical properties of rubber bearings. Accordingly, this study statistically analyzes the third-cycle hysteresis loops of LRB700 bearings under different shear strain amplitudes and temperature conditions. The test results at 0 °C and −20 °C are normalized by those obtained at 23 °C, thereby deriving temperature-dependent modification factors for the corresponding mechanical parameters, as summarized in Table 6.

(4) $$Q_{d}(T)=\frac{Q_{d}(T)}{Q_{d}(23℃)}Q_{d}(23℃)=C_{Q_{d}}(T)Q_{d}(23℃)$$

(5) $$K_{post}(T)=\frac{K_{post}(T)}{K_{post}(23℃)}K_{post}(23℃)=C_{K_{post}}(T)K_{post}(23℃)$$

(6) $$K_{h}=\frac{K_{h}(T)}{K_{h}(23℃)}K_{h}(23℃)=C_{K_{h}}(T)K_{h}(23℃)$$

## 4. Key Factors Affecting the Dynamic Behavior of Lead/Rubber Bearings

Based on the temperature-dependent compression–shear test results of rubber bearings presented above, this section develops a uniaxial constitutive model incorporating the effects of ambient temperature, hysteretic heating of the lead core, and large-strain hardening. A single-degree-of-freedom (SDOF) base-isolated system is then adopted to perform seismic response analyses, aiming to investigate the influence of variations in key dynamic characteristics of rubber bearings under multiple interacting factors on the seismic response of isolated structures.

### 4.1. Uniaxial Constitutive Model for Lead/Rubber Bearings

#### 4.1.1. Overview of the Uniaxial Bouc–Wen Hysteretic Model

**Figure 10:**
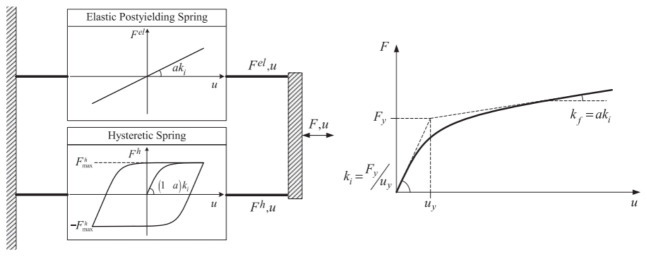
Schematic representation of the Bouc–Wen model.

The Bouc–Wen hysteretic model [6,44,45,46,47], due to its strong versatility and high simulation accuracy, can reproduce a wide range of complex hysteretic behaviors. By adjusting its model parameters, it can account for strength degradation, stiffness degradation, and pinching effects. Consequently, it has been widely applied in structural damage identification, hybrid simulation, and structural health monitoring. A schematic representation of the model is shown in Figure 10.

The classical Bouc–Wen model (without considering stiffness degradation, strength degradation, and pinching effects) is expressed as follows:

(7) $$\dot{z}(t)=\frac{1}{u_{y}}\left[A-|z(t){|}^{n}(\beta +\mathrm{sign}(\bar{u}(t)z(t))\gamma )\right]\bar{u}(t)$$

(8) $$F(t)=F_{el}+F_{h}=\alpha \frac{F_{y}}{u_{y}}u(t)+(1-\alpha )F_{y}z(t)$$

where *α*, *β*, *γ*, *n*, *F_y_* and *uy* are model parameters to be calibrated. *F_el_* and *F_h_* denote the elastic restoring force and the hysteretic restoring force of the structure, respectively.

The Bouc–Wen model parameters for LRB700 under different ambient temperatures at a shear strain amplitude of 100% were identified using the particle swarm optimization (PSO) algorithm [31], and the calibrated results are summarized in Table 7.

**Figure 11:**
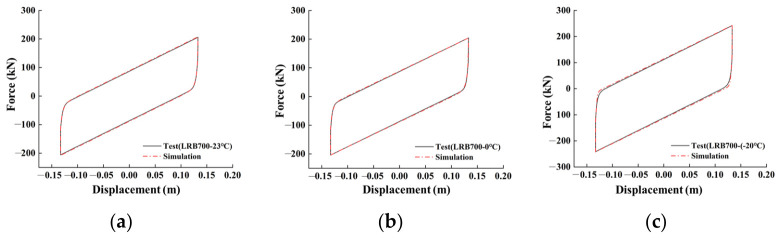
Comparison between the calibrated Bouc-Wen model predictions and the experimental results: (**a**) LRB700-100-23 °C; (**b**) LRB700-100-0 °C; (**c**) LRB700-100-20 °C.

Figure 11 presents a comparison between the calibrated Bouc–Wen model predictions and the experimental results for LRB700. It can be observed that the calibrated model accurately reproduces the hysteretic behavior of the bearings, demonstrating its suitability for subsequent model refinement and further development.

#### 4.1.2. Modified Uniaxial Bouc–Wen Model Considering Ambient Temperature Effects

To account for the influence of ambient temperature, Equation (8) is modified in this section. Based on the experimental data of LRB700 under different temperature conditions, distinct initial values are assigned to the post-yield stiffness and the characteristic strength.

(9) $$\begin{array}{l} \begin{array}{l} F(t)=F_{el}+F_{h}\\ =\alpha \frac{F_{y}}{u_{y}}C_{Kpost}\left(T_{0}\right)u(t)+(1-\alpha )F_{y}C_{Qd}\left(T_{0}\right)z(t) \end{array}\\ =K_{post}C_{Kpost}\left(T_{0}\right)u(t)+Q_{d}C_{Qd}\left(T_{0}\right)z(t) \end{array}$$

where $C_{Kpost}\left(T_{0}\right)$ and $C_{Qd}\left(T_{0}\right)$ denote the modification factors for the post-yield stiffness and the characteristic strength under different ambient temperatures, respectively (as listed in Table 6).

#### 4.1.3. Uniaxial Bouc–Wen Model Considering Lead Core Hysteretic Heating

**Figure 12:**
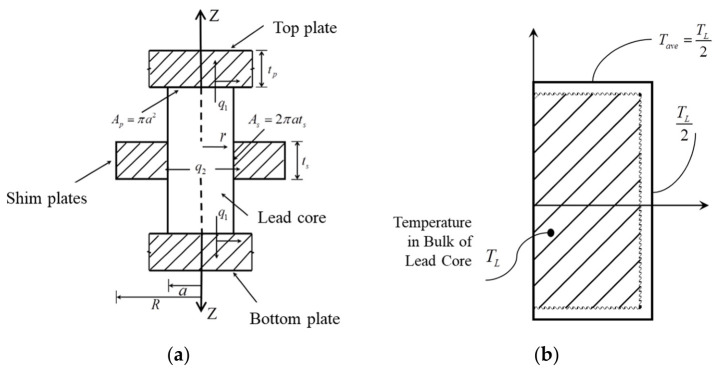
Temperature variation of lead/rubber bearing and lead core: (**a**) model for the analysis of heat conduction in lead/rubber bearings; (**b**) assumed lead core temperature distribution.

According to Constantinou et al. [20], the temperature of the lead core in lead/rubber bearings rises progressively as the number of horizontal loading cycles increases. In the initial stage, the characteristic strength and energy dissipation capacity decrease significantly, followed by a gradual stabilization in the later stage. This behavior can be explained as follows: the plastic work induced by external loading is converted into heat, leading to a temperature increase in the lead core and a corresponding degradation of its mechanical properties. Meanwhile, due to the much lower thermal conductivity of rubber compared to that of steel plates and the lead core, heat is gradually transferred from the lead core to the top and bottom end plates as well as the internal steel layers (as shown in Figure 12). When a thermal equilibrium is reached between heat generation in the lead core and heat dissipation through the surrounding steel components, the temperature of the lead core stabilizes, and the characteristic strength correspondingly approaches a steady state.

The mechanical work performed by external loading on the lead core, expressed per unit time and per unit volume, is given by Equation (10).

(10) $$q^{\prime \prime \prime }(t)=\frac{\left|\sigma_{YL}\cdot A_{L}\cdot \frac{du}{dt}\right|}{V_{L}}=\frac{\sigma_{YL}\cdot \left|\frac{du}{dt}\right|}{h_{L}}$$

where $q^{\prime \prime \prime }(t)$ is the heat flux density; $\sigma_{YL}$ is the yield strength of the lead core; $A_{L}$ is the cross-sectional area of the lead core; $h_{L}$ is the height of the lead core; $V_{L}$ is the volume of the lead core ($V_{L}=A_{L}\cdot h_{L}$).

According to the law of energy conservation, the change in internal energy of the lead core is equal to the mechanical work performed by external loading minus the heat dissipated through the top and bottom end plates as well as the internal steel layers, which can be expressed as follows:

(11) $$\rho_{L}c_{L}V_{L}\frac{dT_{L}}{dt}=q^{\prime \prime \prime }(t)\cdot V_{L}-2\cdot q_{1}(t)-q_{2}(t)$$

where $\rho_{L}$ is the density of the lead core, $c_{L}$ is the specific heat capacity, $q_{1}(t)$ denotes the heat dissipated through the top and bottom end plates at time *t*, $q_{2}(t)$ represents the heat dissipated through the internal steel layers at time t.

Substituting Equations (4)–(6) into Equation (11) yields Equation (12):

(12) $$\begin{array}{l} \frac{dT_{L}}{dt}=\frac{\sigma_{YL}\left(T_{L}\right)\cdot v(t)}{\rho_{L}c_{L}h_{L}}-\frac{k_{S}\cdot T_{L}}{a\cdot \rho_{L}c_{L}h_{L}}\cdot \left(\frac{1}{F}+1.274\cdot \left(\frac{t_{s}}{a}\right)\cdot {\left(t^{+}\right)}^{-1/3}\right)\\ F=\left\{\begin{array}{c} 2\cdot {\left(\frac{t^{+}}{\pi }\right)}^{1/2}-\frac{t^{+}}{\pi }\cdot \left[2-\left(\frac{t^{+}}{4}\right)-{\left(\frac{t^{+}}{4}\right)}^{2}-\frac{15}{4}{\left(\frac{t^{+}}{4}\right)}^{3}\right],t^{+}<0.6\\ \frac{8}{3\pi }-\frac{1}{2{\left(\pi \cdot t^{+}\right)}^{1/2}}\cdot \left[1-\frac{1}{3\cdot \left(4t^{+}\right)}+\frac{1}{6\cdot {\left(4t^{+}\right)}^{2}}-\frac{1}{12\cdot {\left(4t^{+}\right)}^{3}}\right],t^{+}\geq 0.6 \end{array}\right. \end{array}$$

As shown in Figure 12, the boundary temperature of the end plates is $T_{ave}$, and the radius of the lead core is a. To address the heat conduction from the lead core to the end plates, Kalpakidis [25] introduced the following dimensionless parameters to simplify the formulation of the heat transfer at the interface between the lead core and the end plates:

(13) $$t^{+}=\frac{\alpha \cdot t}{\alpha^{2}}$$

where *α* is the thermal diffusivity of the end plates, and *t*^+^ is the dimensionless time corresponding to time t.

Based on the above theoretical analysis, the relationship between the yield strength of the lead core and temperature at any time t can be established. It should be noted that the temperature-dependent relationship for the yield strength of the lead core proposed by Kalpakidis [25] may lead to an excessively rapid temperature rise under long-duration loading conditions, causing the lead core temperature to approach its melting point prematurely. Moreover, even when the lead core reaches its melting point, the yield strength does not reduce to zero. Therefore, in this study, the original relationship between the yield strength of the lead core and temperature is modified as follows:

(14) $$C_{Qd}(T)=\frac{Q_{d}(T)}{Q_{d}(23{}^{\circ}C)}=\frac{\sigma_{YL}(T)}{\sigma_{YL}(23{}^{\circ}C)}=\left\{\begin{array}{cc} 0.4885e^{-0.0153T}+0.6565 & T=T_{L}+T_{0}\leq 23{}^{\circ}C\\ e^{-0.0069(T-23)} & 23{}^{\circ}C<T\leq 250{}^{\circ}C\\ e^{-0.0069(250-23)}-\left(\frac{327-T}{327-250}\right) & 250{}^{\circ}C<T\leq 327{}^{\circ}C\\ 0 & 327{}^{\circ}C<T=T_{L}+T_{0} \end{array}\right.$$

where *T_L_* is the temperature change of the lead core; *T*_0_ is the initial ambient temperature; *T* is the instantaneous temperature of the lead core; and $C_{Qd}(T)$ is the temperature-dependent adjustment factor for the characteristic strength. It should be noted that, due to the relatively low temperature sensitivity of rubber materials, the temperature rise induced by hysteretic heating of the lead core does not significantly alter the mechanical properties of the rubber component within the bearing. Moreover, since the post-yield stiffness of rubber bearings is primarily governed by ambient temperature, the effect of hysteretic heating on post-yield stiffness can be reasonably neglected.

#### 4.1.4. Uniaxial Bouc–Wen Model Considering Large-Strain Hardening

The strain hardening effect of rubber bearings at large deformation is primarily attributed to the hyperelastic behavior of rubber. In the Bouc–Wen model, the linear term *KpostU* is mainly used to represent the mechanical response of the rubber component. Therefore, the large-strain hardening effect can be incorporated by modifying this linear term. To account for strain hardening in high-damping rubber bearings under large deformation, Grant [44] proposed a hyperelastic constitutive formulation in which the strain energy is expressed as a fifth-order polynomial of horizontal shear displacement for a specific shear state. Following this approach, the present study embeds a fifth-order function of horizontal shear displacement into the term *KpostU* to capture the large-strain hardening effect, i.e.,

(15) $$K_{post}U\to K_{post}G(|\gamma |)=K_{post}\left[a_{1}|\gamma |+a_{2}|\gamma {|}^{3}+a_{2}|\gamma {|}^{5}\right]$$

where *a*_1_, *a*_2_, and *a*_3_ are material parameters, with values of *a*_1_ = 0.0015, *a*_2_ = −0.000015, and *a*_3_ = 0.0015, respectively; *γ* denotes the shear strain, defined as the ratio of horizontal displacement to the total thickness of the rubber layers in the rubber bearing.

### 4.2. Key Factors Affecting the Dynamic Behavior of Lead/Rubber Bearings: An SDOF Base-Isolated System Study

**Figure 13:**
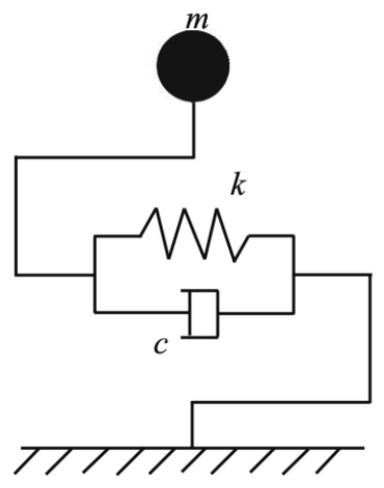
Single-degree-of-freedom base-isolated system model for LRB700.

After establishing the uniaxial constitutive model of lead/rubber bearings incorporating the effects of ambient temperature, lead core hysteretic heating, and large-strain hardening, the dynamic time history response of isolated structures under seismic excitation can be investigated. Due to the presence of the isolation layer, the superstructure above the isolation level predominantly undergoes rigid-body translation during earthquakes. Therefore, for low-rise isolated structures, the mechanical system can be simplified as a single-degree-of-freedom (SDOF) model consisting of a lumped mass, a linear spring, and a viscous damper, as illustrated in Figure 13.

Neglecting the P–Δ effect induced by gravity and vertical ground acceleration, the governing equation of motion for the SDOF base-isolated system subjected to unidirectional horizontal seismic excitation can be expressed as follows:

(16) $$m\ddot{u}+c\dot{u}+F(t)=-m{\ddot{u}}_{g}$$

where *c* denotes the damping of the isolation layer; F(t) is the restoring force of the isolation layer, which can be represented by the modified Bouc–Wen model mentioned above; *u* is the horizontal displacement of the superstructure; and ${\ddot{u}}_{g}$ denotes the ground acceleration. In the SDOF base-isolated system, the mass of the superstructure (m) is taken as the equivalent mass corresponding to the LRB700 bearing subjected to a vertical compressive stress of 15 MPa, which is 577 tons. The governing differential equation can be solved using the Newmark-*β* method.

**Figure 14:**
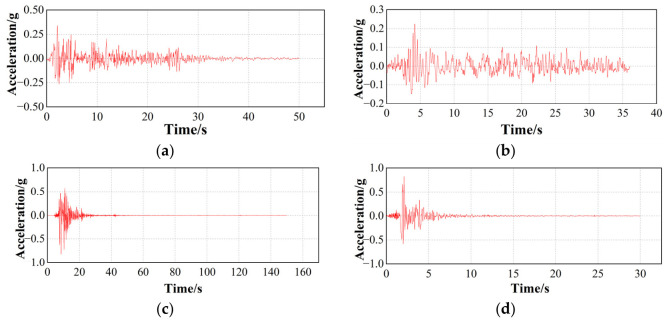
Acceleration time histories: (**a**) El Centro (1940) Ground Motion; (**b**) Hachinohe (1968) Ground Motion; (**c**) Kobe (1995) Ground Motion; (**d**) Northridge (1994) Ground Motion.

To investigate the effects of ambient temperature and lead core hysteretic heating on the dynamic response of base-isolated systems, this section conducts time history analyses on an SDOF base-isolated system under different temperature conditions. Four ground motion records are considered, including two near-fault earthquakes (Northridge, 1994 and Kobe, 1995) and two far-field earthquakes (El Centro, 1940 and Hachinohe, 1968), as shown in Figure 14. These records are first normalized and subsequently scaled to target intensity levels, to evaluate the response of the SDOF base-isolated system under design and rare earthquake scenarios corresponding to an intensity of 8 (0.3 g).

In this section, a total of 36 seismic response analyses were performed for the SDOF base-isolated system under three temperature conditions: 23 °C, 0 °C, and −20 °C. Due to space limitations, only the results corresponding to the Hachinohe (1968) ground motion are presented as a representative example. For clarity, in all comparison figures, black, red, and blue curves represent the results at 23 °C, 0 °C, and −20 °C, respectively.

#### 4.2.1. Ambient Temperature Effects

**Figure 15:**
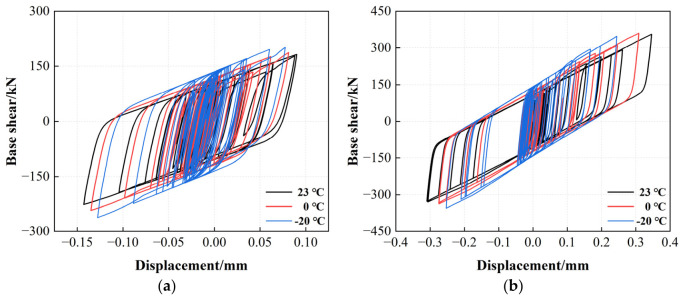
Hysteretic curves of SDOF base-isolated system at different temperatures: (**a**) at 0.68 m/s^2^ peak acceleration; (**b**) at 1.20 m/s^2^ peak acceleration.

Figure 15 compares the hysteretic responses of the SDOF base-isolated system under the Hachinohe (1968) ground motion at different ambient temperatures. It can be observed that, as the ambient temperature decreases, the hysteresis loops become narrower and fuller, with an increase in characteristic strength and a reduction in hysteretic displacement.

Table 8 and Table 9 summarize and normalize the seismic responses of an SDOF base-isolated system under design earthquakes at different ambient temperatures, with the peak responses at 23 °C set as the benchmark. The mean responses for far-field and near-field ground motions are averaged from typical earthquake records. As the ambient temperature drops to 0 °C and −20 °C, the increased bearing stiffness raises the base shear and reduces the isolation layer displacement under both near- and far-field excitations. Relative to 23 °C, the base shear rises by 7.5% and 15% at 0 °C and −20 °C, while the isolation displacement decreases by 6% and 10%, respectively.

Table 10 and Table 11 respectively list the seismic responses of the SDOF base-isolated system and their normalized values under the maximum considered earthquakes at various ambient temperatures, with the peak responses at 23 °C serving as the reference benchmark. With the drop in ambient temperature to 0 °C and −20 °C, the rising stiffness of rubber bearings leads to higher base shear and lower isolation layer displacement under both near-field and far-field ground motions. Relative to the 23 °C condition, the base shear rises by 5% and 10% at 0 °C and −20 °C, whereas the isolation displacement reduces by 9% and 13%, respectively.

#### 4.2.2. Lead Core Hysteretic Heating Effects

**Figure 16:**
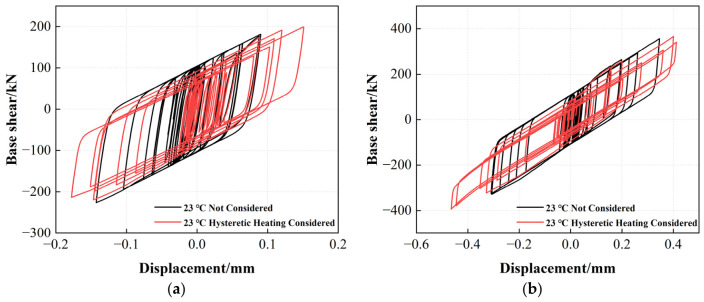
Comparison of hysteretic curves for SDOF base-isolated system at 23 °C with and without hysteretic heating: (**a**) at 0.68 m/s^2^ peak acceleration; (**b**) at 1.20 m/s^2^ peak acceleration.

Figure 16 compares the hysteretic responses of the SDOF base-isolated system under the Hachinohe (1968) ground motion at an ambient temperature of 23 °C, with and without consideration of the lead core hysteretic heating effect. It can be observed that incorporating hysteretic heating significantly increases the isolation layer displacement while reducing the horizontal characteristic strength of the bearing. This effect becomes more pronounced under the maximum considered earthquake conditions.

Table 12 and Table 13 summarize the seismic responses of the SDOF base-isolated system under design-level earthquakes considering lead core hysteretic heating, along with the corresponding normalized results. The normalization adopts the peak responses without hysteretic heating at the same temperature as the baseline. The results show that accounting for hysteretic heating evidently amplifies the isolation layer displacement, with a more remarkable effect under far-field seismic excitations. Specifically, the isolation displacement rises by 15%, 11% and 14% at 23 °C, 0 °C and −20 °C, respectively. By contrast, the base shear is dominated by the coupling of isolation displacement and lead core yield strength, and is closely related to seismic intensity and lead core temperature. Overall, the base shear remains basically stable at all temperatures under design-level near-field and far-field ground motions.

Table 14 summarizes the seismic response of an SDOF base-isolated system under the maximum considered earthquakes, accounting for lead core hysteretic heating at different ambient temperatures. Including this effect reduces the isolation system’s base shear but increases isolation layer displacement. Table 15 normalizes these results relative to the no-heating case at the same temperatures. The heating effect raises displacement by 13% at 23 °C, 9% at 0 °C, and 11% at −20 °C. Base shear, influenced jointly by displacement and lead core yield strength, declines by 7.5%, 9%, and 13% at the three respective temperatures.

#### 4.2.3. Coupled Effects of Ambient Temperature and Lead Core Hysteretic Heating

As discussed above, the hysteretic heating effect of the lead core primarily leads to an increase in isolation layer displacement and a reduction in base shear, whereas low ambient temperatures tend to decrease isolation layer displacement and increase base shear. To investigate the coupled effects of ambient temperature and lead core hysteretic heating on the seismic response of isolation systems, a comparative analysis is conducted between the responses of the SDOF base-isolated system under ambient conditions without considering hysteretic heating and those under different temperature conditions with hysteretic heating considered.

Table 16 shows the normalized seismic responses of the isolation system considering hysteretic heating, referenced to the 23 °C case without heating. Under design-level earthquakes, the governing effect is temperature-dependent: at 23 °C, hysteretic heating dominates, increasing isolation displacement by 8.5% and reducing base shear by 4%; at −20 °C, ambient temperature governs, decreasing isolation displacement by 5% and increasing base shear by 15%; at 0 °C, the two effects combine, leading to a 2.5% reduction in isolation displacement and a 6% increase in base shear.

Table 17 shows the normalized seismic responses (referenced to the non-heating case at the same temperature) under the maximum considered earthquakes. The governing effect is temperature-dependent: at 23 °C, hysteretic heating dominates, increasing isolation displacement by 13% and reducing base shear by 6.5%; at −20 °C, ambient temperature governs, decreasing isolation displacement by 5% and increasing base shear by 7%; at 0 °C, the two effects combine, leading to a 3% reduction in isolation displacement and a 5% increase in base shear.

## 5. Conclusions

This study systematically investigates the thermo-mechanical behavior of lead/rubber bearings (LRBs) by considering the coupled effects of ambient temperature and lead core hysteretic heating. Dynamic cyclic loading tests are conducted on full-scale LRBs (700 mm in diameter) under a wide range of ambient temperatures. Then, a modified uniaxial Bouc–Wen constitutive model is proposed, incorporating ambient temperature effects, hysteretic heating, and large-strain hardening. Finally, seismic analyses of an SDOF base-isolated system are conducted to evaluate the performance of isolation systems under complex service conditions. Based on temperature-dependent dynamic compression–shear tests and subsequent numerical analyses, the following conclusions can be drawn:

(1) Ambient temperature effect: Ambient temperature significantly influences the mechanical properties of LRBs. Through detailed mechanism analysis of the low-temperature test results and in-depth discussion of the numerical simulation results, it is found that as temperature decreases from 23 °C to −20 °C, the characteristic strength and equivalent stiffness increase by approximately 32% and 24%, respectively, resulting in reduced isolation layer displacement (by 10–13%) but amplified base shear (by 10–15%). This indicates that low-temperature conditions may adversely increase force demand on superstructures, and the underlying mechanism is closely related to the change in rubber viscoelasticity and lead core plastic deformation capacity under low-temperature environments, which is also verified by the refined numerical simulation results.
(2) Hysteretic heating effect: The hysteretic heating of the lead core induces a progressive temperature rise during cyclic loading, leading to degradation of characteristic strength and stiffness. Combined with the refined numerical simulation analysis, it is confirmed that isolation layer displacement increases by approximately 11–13%, while base shear decreases by 2–10%, with more pronounced effects under the maximum considered earthquake conditions. The refined discussion further clarifies the correlation between the degree of hysteretic heating, loading duration and the degradation extent of bearing mechanical properties, which supplements the depth of the conclusion.
(3) Coupled temperature effects and competing mechanism: Ambient temperature and hysteretic heating exhibit a clear competing mechanism. Focusing on the coupled effect of the two, this study deeply analyzes their combined influence law on the mechanical properties of isolation bearings and the response of base-isolated structures: at low temperatures, ambient temperature dominates, resulting in increased stiffness and reduced displacement; at moderate temperatures, both effects interact, and their comprehensive influence is closely related to loading frequency and amplitude; whereas at higher temperatures or under long-duration strong motions, hysteretic heating becomes dominant, leading to strength degradation and displacement amplification. This refined analysis further improves the logic of the conclusion and clarifies the internal mechanism of the coupled temperature effect.

Overall, this study highlights the critical role of coupled temperature effects in governing the dynamic behavior of LRBs. The results suggest that current design provisions may be insufficient to capture the full response evolution under combined temperature effects. Future design methodologies should incorporate both initial temperature conditions and in situ temperature rise due to cyclic loading.

## Figures and Tables

**Table 1:** The geometric dimensions of the LRB700 specimen.

Main Parameter	LRB700
Diameter of the bearing/mm	720.00
Lead diameter/mm	104.20
Diameter of the cover plate/mm	990.00
Thickness of the rubber sheet/mm	7.39
Number of the rubber sheet	18
Thickness of the steel shim/mm	4.00
Number of the steel sheet	17
Total thickness of the rubber/mm	133.02
Height of the bearing/mm	271.02

**Table 2:** Loading conditions of the LRB700 specimen.

Vertical Pressure/ MPa	Vertical Force/ kN	Loading Frequency/ Hz	Ambient Temperature/ °C	Shear Strain/ %	Horizontal Displacement/ mm	Peak Velocity/(mm s^−1^)
15	5770	0.20	23/0/−20	50	66.5	83.5
				100	133.0	167.0
				250	332.5	417.6
			23/0/−20	50	66.5	104.4
				100	133.0	208.8
				250	332.5	522.0
			23/0/−20	50	66.5	125.3
				100	133.0	250.6
				250	332.5	626.4

**Table 3:** Characteristic strength of LRB700 bearings at the third cycle.

Performance Parameter	Ambient Temperature (°C)	LRB700-1	LRB700-2	LRB700-3	Average Value	Change Percentage
Characteristic strength/kN	−20	114.64	131.91	101.13	115.89	+32%
	0	96.12	114.94	91.16	100.74	+15%
	23	77.18	102.81	83.61	87.86	-

**Table 4:** Post-yield stiffness of LRB700 bearings at the third cycle.

Performance Parameter	Ambient Temperature (°C)	LRB700-1	LRB700-2	LRB700-3	Average Value	Change Percentage
Post-yield stiffness kN/mm	−20	1.01	0.98	0.89	0.96	+13%
	0	0.91	0.94	0.84	0.90	+6%
	23	0.87	0.90	0.78	0.85	-

**Table 5:** Horizontal equivalent stiffness of LRB700 bearings at the third cycle.

Performance Parameter	Ambient Temperature (°C)	LRB700-1	LRB700-2	LRB700-3	Average Value	Change Percentage
Horizontal equivalent stiffness kN/mm	−20	1.93	2.26	1.93	2.04	+24
	0	1.75	1.96	1.78	1.83	+1
	23	1.56	1.79	1.61	1.65	-

**Table 6:** Temperature adjustment coefficient for the LRB700 bearing.

Bearing Type	The Adjustment Coefficient of Characteristic Strength	The Adjustment Coefficient of Post-Yield Stiffness	The Adjustment Coefficient of Horizontal Equivalent Stiffness
LRB700	$C_{Q_{d}}(T)=0.4885e^{-0.0153T}+0.6565$	$C_{K_{post}}(T)=1.0630e^{-0.0029T}$	$C_{K_{h}}(T)=1.1210e^{-0.0048T}$

**Table 7:** The calibrated parameters of LRB700.

Case	*β*	*γ*	*n*	*α*	*F_y_*/kN	*u_y_*/m
23 °C-LRB700-100%	−1.000	2.000	0.321	0.048	91.367	0.004
0 °C-LRB700-100%	−1.000	2.000	0.236	0.034	93.000	0.003
−20 °C-LRB700-100%	−0.977	1.977	0.140	0.027	96.437	0.002

**Table 8:** Performance index under ambient temperature effects at 0.68 m/s^2^ peak acceleration.

Performance Index	Near-Field			Far-Field		
	23 °C	0 °C	−20 °C	23 °C	0 °C	−20 °C
Displacement (m)	0.061	0.058	0.055	0.116	0.109	0.103
Base shear (kN)	144.194	155.396	166.454	202.664	216.801	233.186

**Table 9:** Normalized results of performance index under ambient temperature effects at 0.68 m/s^2^ peak acceleration.

Performance Index	Near-Field			Far-Field		
	23 °C	0 °C	−20 °C	23 °C	0 °C	−20 °C
Displacement (m)	-	0.948	0.901	-	0.944	0.891
Base shear (kN)	-	1.078	1.154	-	1.070	1.151

**Table 10:** Performance index under ambient temperature effects at 1.20 m/s^2^ peak acceleration.

Performance Index	Near-Field			Far-Field		
	23 °C	0 °C	−20 °C	23 °C	0 °C	−20 °C
Displacement (m)	0.116	0.105	0.098	0.260	0.240	0.231
Base shear (kN)	197.605	207.520	211.229	301.732	314.820	339.839

**Table 11:** Normalized results of performance index under ambient temperature effects at 1.20 m/s^2^ peak acceleration.

Performance Index	Near-Field			Far-Field		
	23 °C	0 °C	−20 °C	23 °C	0 °C	−20 °C
Displacement (m)	-	0.903	0.848	-	0.923	0.890
Base shear (kN)	-	1.050	1.069	-	1.043	1.126

**Table 12:** Performance index considering hysteretic heating effect of lead core under the peak acceleration of 0.68 m/s^2^.

Performance Index	Near-Field			Far-Field		
	23 °C	0 °C	−20 °C	23 °C	0 °C	−20 °C
Displacement (m)	0.062	0.061	0.058	0.134	0.121	0.117
Base shear (kN)	137.132	153.297	162.860	196.926	214.901	236.374

**Table 13:** Normalized results of performance index considering hysteretic heating effect of lead core under the peak acceleration of 0.68 m/s^2^.

Performance Index	Near-Field			Far-Field		
	23 °C	0 °C	−20 °C	23 °C	0 °C	−20 °C
Displacement (m)	1.024	1.052	1.057	1.154	1.107	1.135
Base shear (kN)	0.951	0.986	0.978	0.972	0.991	1.014

**Table 14:** Performance index considering hysteretic heating effect of lead core under the peak acceleration of 1.20 m/s^2^.

Performance Index	Near-Field			Far-Field		
	23 °C	0 °C	−20 °C	23 °C	0 °C	−20 °C
Displacement (m)	0.114	0.110	0.108	0.321	0.271	0.257
Base shear (kN)	187.798	191.781	211.405	278.435	284.282	293.998

**Table 15:** Normalized results of performance index considering hysteretic heating effect of lead core under the peak acceleration of 1.20 m/s^2^.

Performance Index	Near-Field			Far-Field		
	23 °C	0 °C	−20 °C	23 °C	0 °C	−20 °C
Displacement (m)	0.982	1.049	1.096	1.236	1.131	1.113
Base shear (kN)	0.950	0.924	1.001	0.923	0.903	0.865

**Table 16:** Normalized results of performance index under the peak acceleration of 0.68 m/s^2^.

Performance Index	Near-Field			Far-Field		
	23 °C	0 °C	−20 °C	23 °C	0 °C	−20 °C
Displacement (m)	1.024	0.997	0.952	1.154	1.045	1.012
Base shear (kN)	0.951	1.063	1.129	0.972	1.060	1.166

**Table 17:** Normalized results of performance index under the peak acceleration of 1.20 m/s^2^.

Performance Index	Near-Field			Far-Field		
	23 °C	0 °C	−20 °C	23 °C	0 °C	−20 °C
Displacement (m)	0.982	0.947	0.929	1.236	1.044	0.990
Base shear (kN)	0.950	0.971	1.070	0.923	0.942	0.974

## Data Availability

The original contributions presented in this study are included in the article. Further inquiries can be directed to the corresponding author.
